# Exploring the molecular link between obstructive sleep apnea and interstitial cystitis/bladder pain syndrome: A bioinformatics and machine learning study

**DOI:** 10.1371/journal.pone.0339824

**Published:** 2025-12-31

**Authors:** Yang Xu, Fei Jiang, Bin Zheng, Guang-Lei Zhang, Ren-Hu Li

**Affiliations:** 1 Department of Anesthesiology, Lu’an Hospital of Anhui Medical University, Lu’an, Anhui, China; 2 Department of Pain, Xiangyang Central Hospital, Affiliated Hospital of Hubei University of Arts and Science, Xiangyang, Hubei, China; 3 Department of Anesthesiology, Xiangyang Central Hospital, Affiliated Hospital of Hubei University of Arts and Science, Xiangyang, Hubei, China; Xuzhou Central Hospital, The Xuzhou School of Clinical Medicine of Nanjing Medical University, CHINA

## Abstract

**Background:**

Obstructive sleep apnea (OSA) and interstitial cystitis/bladder pain syndrome (IC/BPS) are chronic conditions that significantly impact patients’ quality of life. OSA involves recurrent upper airway obstruction during sleep, causing hypoxia and fragmented sleep linked to cardiovascular and metabolic issues. IC/BPS is defined by chronic pelvic pain and urinary symptoms; its pathophysiology is complex and poorly understood. The overlap in the prevalence of OSA and IC/BPS suggests a possible shared pathophysiological link. This study aimed to identify shared molecular mechanisms and diagnostic biomarkers between OSA and IC/BPS through integrated bioinformatics approaches.

**Methods:**

This study used bioinformatics and machine learning to analyze transcriptomic data for OSA and IC/BPS, identifying differential expressed genes (DEGs) and enriched pathways from Gene Expression Omnibus (GEO) database. Weighted gene co-expression network analysis (WGCNA) constructed gene co-expression networks and identified hub genes, while immune infiltration analysis characterized the immune microenvironment. Four machine learning algorithms developed diagnostic models and also identified key markers.

**Results:**

A total of 2,233 DEGs were identified in OSA and 1,183 in IC/BPS, with 93 overlapping genes. Among these, machine learning algorithms identified DUSP9 as the single common gene linking both disorders, forming two-gene signatures for each condition (*DUSP9*/*CCDC68* for OSA and *DUSP9*/*KPNA2* for IC/BPS). Key pathways for OSA included RIG-I-like and NOD-like receptor signaling. In contrast, IC/BPS was linked to cytokine interactions and JAK-STAT signaling. Immune infiltration analysis showed that *DUSP9* expression was correlated with CD56dim natural killer cells in OSA and with activated CD4 T cells in IC/BPS, further supporting its role in the immune response associated with these disorders.

**Conclusions:**

This study established *DUSP9* as a pivotal shared biomarker and central regulator linking OSA and IC/BPS through integrated bioinformatics analysis.

## 1 Introduction

Obstructive sleep apnea (OSA) and interstitial cystitis/bladder pain syndrome (IC/BPS) are long-lasting disorders that significantly impact patients’ health and quality of life. OSA is characterized by recurrent episodes of upper airway obstruction during sleep. These episodes lead to hypoxia and sleep fragmentation, which are linked to cardiovascular diseases and metabolic disorders [1]. IC/BPS is marked by chronic pelvic pain and urinary symptoms. It poses a complex clinical challenge due to its incompletely understood pathophysiology [2]. The potential overlap in the prevalence of OSA and IC/BPS, as demonstrated by epidemiological evidence [3], indicates an association that may reflect shared pathophysiological features, though the specific mechanisms require further investigation. Therefore, understanding these mechanisms is crucial for developing integrated treatment approaches that could improve patient outcomes and reduce the burden of these conditions.

Despite the recognized clinical importance of OSA and IC/BPS, the molecular interactions between these conditions are not well characterized. Theoretical connections suggest shared biological processes, such as systemic inflammation and neuroendocrine dysregulation, which are central to the pathogenesis of both conditions [4]. For instance, intermittent hypoxia and sleep fragmentation in OSA could lead to systemic inflammation. These changes may disrupt the hypothalamic-pituitary-adrenal (HPA) axis, resulting in increased cortisol levels and insulin resistance [5]. Moreover, the activation of the sympathetic nervous system and the release of pro-inflammatory cytokines, including tumor necrosis factor-alpha (TNF-α) and interleukin-6 (IL-6), may drive the development and progression of both OSA and IC/BPS [6,7]. However, concrete molecular evidence supporting these connections is lacking. Existing knowledge mainly relies on symptom correlations, without clear genetic or transcriptomic signatures defining their relationship. This gap in understanding hinders the development of targeted therapies and personalized treatment plans. Therefore, advanced bioinformatics approaches are urgently needed to explore the molecular basis of the association between OSA and IC/BPS. Such research could uncover new biomarkers and therapeutic targets, significantly advancing the field.

This study aims to leverage integrated bioinformatics and machine learning approaches to analyze transcriptomic data relevant to OSA and IC/BPS. Our objectives are to: (1) identify differentially expressed genes and enriched biological pathways common to both conditions; (2) explore protein interaction networks for potential hub genes; (3) develop diagnostic models using machine learning algorithms; and (4) characterize features of the immune microenvironment associated with candidate genes. These analyses aim to provide preliminary molecular insights into the relationship between OSA and IC/BPS, which may lead to new diagnostic and therapeutic strategies to improve patient care and outcomes.

## 2 Methods

### 2.1 Datasets and data processing

**Fig 1:**
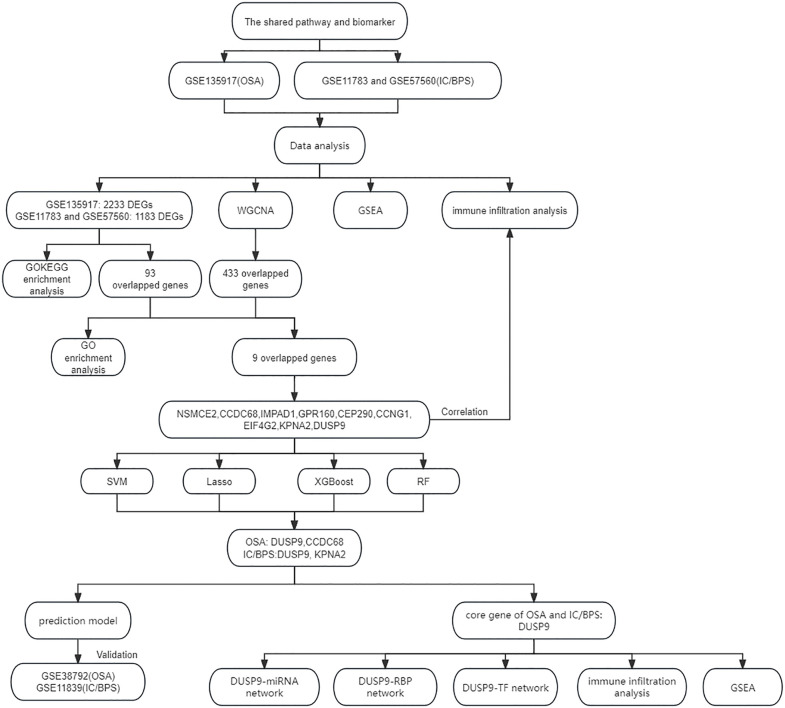
Workflow diagram.

Fig 1 illustrates the basic research workflow. The OSA datasets (GSE135917) and two IC/BPS datasets (GSE11783 and GSE57560) from Gene Expression Omnibus (GEO) (https://www.ncbi.nlm.nih.gov/geo/) were analyzed (S1 and S2 Tables in S1 File). Both GSE135917 and GSE38792 include samples from 8 controls and 10 OSA patients, and both datasets use adipose tissue. GSE135917 was used for initial discovery analyses, while GSE38792 served for external validation. For IC/BPS, GSE11783 and GSE57560 were merged, resulting in 9 controls and 23 IC/BPS patients, while GSE11839 included 6 controls and 6 IC/BPS patients. All IC/BPS samples were derived from bladder tissue. All annotation and data extraction were performed using R (version 4.2.1, https://www.r-project.org/). Subsequently, differentially expressed genes (DEGs) in OSA and IC/BPS were identified by comparing experimental and control groups using thresholds of P < 0.05 and |log2FC| > 0. This initial permissive threshold was employed to maximize sensitivity in the discovery phase. To validate robustness and address potential concerns regarding threshold stringency, we tested stricter criteria including |log2FC| > 0.58. Based on these evaluations, a |log2FC| > 0.3 threshold was selected for the final integrated analysis as it provided an optimal balance between statistical rigor and sufficient features for downstream machine learning applications.

### 2.2 Gene ontology and Kyoto encyclopedia of genes and genomes enrichment analysis

Gene Ontology (GO) enrichment analysis extracts biological information from genetic data, while Kyoto Encyclopedia of Genes and Genomes (KEGG) pathway analysis helps to understand biological functions. OSA and IC/BPS gene sets and expression matrices were analyzed to identify regulatory pathways using GO and KEGG analyses.

### 2.3 Construction and module analysis of weighted gene co-expression network analysis

We used the “WGCNA” package in R to build a gene co-expression network. To ensure data reliability, we retained samples with an average FPKM above 0.5. Samples were clustered using “flashClust.” We determined the optimal soft threshold for a scale-free network with “pickSoftThreshold” and converted the adjacency matrix to a Topological Overlap Matrix (TOM). For module detection, we set the cut height to 0.2. The minimum module size was set to 80 for OSA and 50 for IC/BPS. We then calculated module membership and gene significance. Finally, we identified modules linked to OSA and IC/BPS and intersected their genes with DEGs for further analysis.

### 2.4 Gene set enrichment analysis enrichment analysis

The gene set enrichment analysis (GSEA) method ranks genes by phenotype significance and analyzes their distribution in predefined sets, thereby clarifying their enrichment in relevant biological processes.

### 2.5 Immune infiltration analysis

We used single-sample gene enrichment analysis (ssGSEA) with the ‘GSVA’ R package to analyze the infiltration of 28 immune cell types. In this study, we employed the ssGSEA algorithm to assess the relative infiltration abundance and correlation of immune cells in the OSA and normal groups, as well as in the IC/BPS and normal groups, followed by visualization of the results.

### 2.6 Machine learning

To identify key markers of OSA and IC/BPS, we applied four machine learning algorithms: Support Vector Machine Recursive Feature Elimination (SVM-RFE), Least Absolute Shrinkage and Selection Operator Logistic Regression (LASSO), Extreme Gradient Boosting (XGBoost), and Random Forest (RF). Notably, in this study, we evaluated the predictive performance of both LASSO regression using 10-fold cross-validation and SVM-RFE algorithm with 5-fold cross-validation. Additionally, for the RF algorithm, genes with importance scores above 1.0 were considered key markers. For the XGBoost algorithm, we selected the top 10 genes based on their importance as key markers. The intersection of genes filtered by these four algorithms determined the feature genes.

### 2.7 Establishing and validating the nomogram for OSA and IC/BPS

Based on these feature genes, we developed a clinical prediction model for OSA and IC/BPS using the “ggplot2” software package. We evaluated the model’s predictive accuracy using calibration curves and its clinical utility via decision curve analysis. In addition, we assessed each feature gene’s predictive performance in the training and validation sets using receiver operating characteristic (ROC) curves. A larger area under the receiver operating characteristic curve (AUC) indicates better model predictive performance. Finally, we tested the prediction model’s generalizability using the GSE38792 dataset for OSA and the GSE11839 dataset for IC/BPS as validation sets.

### 2.8 mRNA-miRNA, mRNA-RBP and mRNA-TF network construction

From the Starbase database (https://rnasysu.com/encori/), miRNAs and RBPs linked to key genes were extracted, followed by construction of a regulatory network illustrating mRNA-miRNA and mRNA-RBP interactions. Using the ChIPBase database (https://rnasysu.com/chipbase3/index.php), we identified transcription factors that bind to key genes and have created an interaction network. We visualized the networks using Cytoscape software.

## 3 Results

### 3.1 Differential gene screening

**Fig 2:**
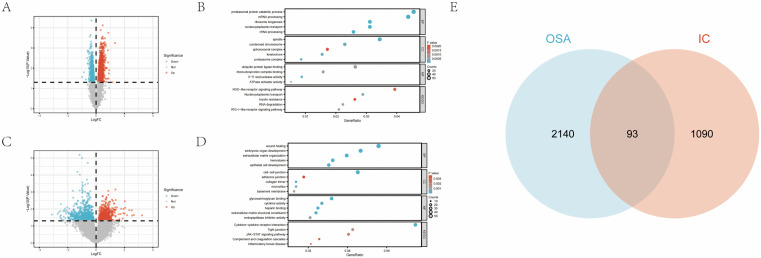
Differential genetic screening. **(A)** Volcano plot of DEGs in GSE135917. **(B)** The GO and KEGG analysis of DEGs in GSE135917. **(C)** Volcano plot of DEGs in GSE11783 and GSE57560. **(D)** The GO and KEGG analysis of DEGs in GSE11783 and GSE57560. **(E)** Venn diagram of DEGs from GSE11783, GSE57560 and GSE135917.

We integrated the GSE11783 and GSE57560 datasets to analyze gene expression profiles in IC/BPS. This resulted in a cohort of 23 IC/BPS samples and 9 control samples. Subsequently, we proceeded to identify DEGs in two distinct datasets. We identified 2,233 DEGs in the GSE135917 dataset and 1,183 DEGs in the combined IC/BPS datasets (S3 Table in S1 File). Volcano plots illustrated their distribution (Fig 2A and 2C), and we identified 93 overlapping genes (Fig 2E). To address potential concerns regarding the sensitivity of our differential expression threshold, we performed a supplementary analysis using a stricter cutoff (|log2FC| > 0.3 and P < 0.05). This approach yielded 822 DEGs in OSA and 575 DEGs in IC/BPS, which remained sufficient for downstream network and machine learning analyses (S4 Table in S1 File).

### 3.2 GO/KEGG enrichment analysis of DEGs

The DEGs in OSA and IC/BPS were subjected to GO and KEGG analyses (S5 Table in S1 File). The GO analysis of OSA revealed several important processes, including mRNA processing, proteasome-mediated protein catabolic process, and nucleocytoplasmic transport. The KEGG analysis identified several key pathways, including the RIG-I-like receptor signaling pathway and the NOD-like receptor signaling pathway (Fig 2B). The KEGG analysis of IC/BPS revealed pathways such as cytokine-

cytokine receptor interaction, JAK-STAT signaling pathway, and pathways related to inflammatory bowel disease (Fig 2D). These results suggested that inflammatory processes may contribute to the pathophysiology of both OSA and IC/BPS.

### 3.3 WGCNA and identify shared genes and shared pathways

**Fig 3:**
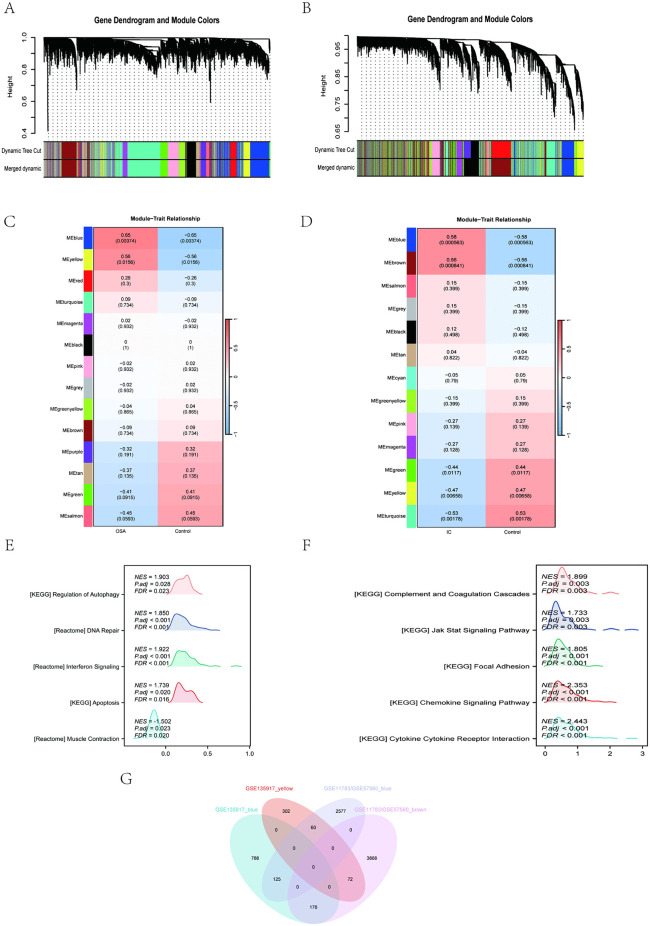
WGCNA and identifying shared genes and shared pathways. **(A)** The cluster dendrogram of co-expression in OSA. **(B)** The cluster dendrogram of co-expression in IC/BPS. **(C)** Correlation between modules and clinical traits in OSA. **(D)** Correlation between modules and clinical traits in IC/BPS. **(E)** Results of the GSEA analysis in OSA. **(F)** Results of the GSEA analysis in IC/BPS. **(G)** Venn diagram showing the overlap of 433 genes in the OSA and IC/BPS modules.

WGCNA was used to identify co-expressed gene clusters that showed differential expression between OSA and IC/BPS. It was also used to calculate the correlation between these modules and disease characteristics. Based on the approximate scale-free topology criterion, the soft threshold β was set to 12 in the OSA model and to 5 in the IC/BPS model (S1 and S2 Figs in S1 File). The co-expression clustering dendrograms of OSA and IC/BPS are shown in Fig 3A and 3B. After merging similar gene modules, two modules were identified in the OSA model (Fig 3C), and two modules were identified in the IC/BPS model (Fig 3D). In both models, the blue module showed the strongest positive correlation with the respective disease, with R = 0.65 for OSA and R = 0.58 for IC/BPS.

We then performed GSEA on OSA and IC/BPS samples to investigate their shared molecular mechanisms (S6 Table in S1 File). In the OSA samples, immune response-related gene sets like Reactome Interferon Signaling (NES = 1.922, FDR < 0.001) were enriched (Fig 3E); similarly, IC/BPS samples were enriched in KEGG Complement and Coagulation Cascades (NES = 1.899, FDR = 0.003) (Fig 3F). Collectively, these results indicate that the immune response plays a crucial role in the shared pathogenic process of OSA and IC/BPS.

**Fig 4:**
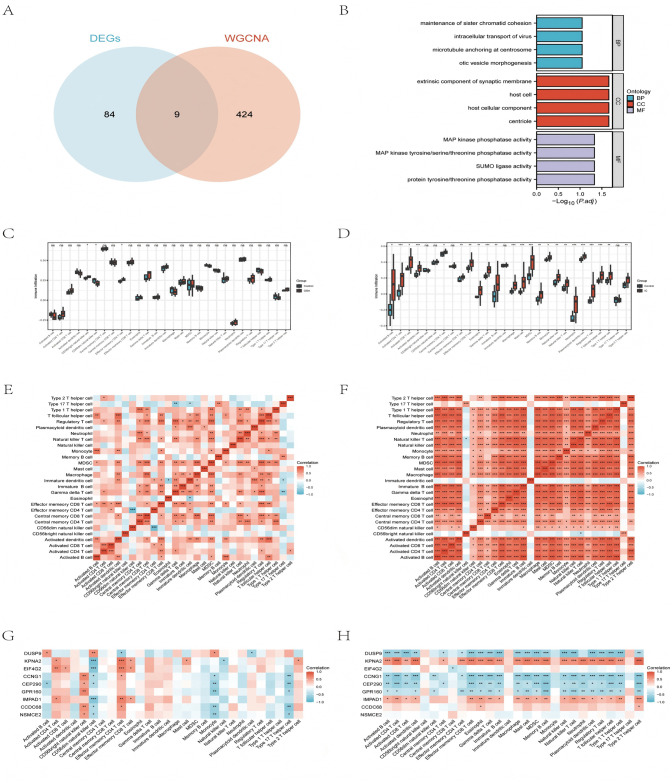
Analysis of immune infltration associated with OSA and IC/BPS. **(A)** The Venn diagram shows that 9 key genes are overlapping between the genes screened by WGCNA and those screened by DEGs. **(B)** Bar map of GO analysis of 9 shared genes between OSA and IC/BPS. **(C)** Box plot of group comparison of immune cell composition of OSA. **(D)** Box plot of group comparison of immune cell composition of IC/BPS. **(E)** Heat map of cell type correlation matrix of OSA. **(F)** Heat map of cell type correlation matrix of IC/BPS. **(G)** Heat map of the correlation between biomarkers and immune cell types of OSA. **(H)** Heat map of the correlation between biomarkers and immune cell types of IC/BPS. * represents p < 0.05, ** represents p < 0.01, *** represents p < 0.001.

There are 433 overlapping genes between the strongest positive modules of OSA and IC/BPS (Fig 3G) (S7 Table in S1 File), possibly related to their pathogenesis. We intersected the 433 WGCNA overlapping genes with 93 differential genes and obtained 9 key genes (Fig 4A) (S8 Table in S1 File). GO analysis of 9 key genes revealed significant enrichment in the molecular function category of MAP kinase phosphatase activity (Fig 4B) (S9 Table in S1 File). This finding highlights the crucial role of the MAPK signaling pathway in regulating cell behavior and disease progression.

### 3.4 Immune infiltration analysis

We analyzed immune cell infiltration characteristics in both OSA patients and healthy controls. Using the ssGSEA algorithm, we identified significant differences in the proportions of CD56bright and CD56dim natural killer cells, as well as effector memory CD4 T cells, between the two groups (Fig 4C). The correlations between immune cells are shown in the Fig 4E. Correlation analysis also showed that *KPNA2*, *EIF4G2*, and *IMPAD1* were positively correlated with activated CD4 T cells, effector memory CD4 T cells, and effector memory CD8 T cells. Conversely, *CCNG1*, *CEP290*, *CCDC68*, and *NSMCE2* were negatively correlated with CD56dim natural killer cells, monocytes, and type 17 T helper cells (Fig 4G). Similarly, we performed a comprehensive analysis of immune cell infiltration between the IC/BPS and healthy control groups. Significant differences were observed in the proportions of immune cells, including activated CD4 T cells, activated dendritic cells, gamma delta T cells, macrophages, mast cells, myeloid-derived suppressor cells (MDSCs), neutrophils, plasmacytoid dendritic cells, regulatory T cells, and T follicular helper cells (Fig 4D). The correlations among immune cells are shown in Fig 4F. Correlation analysis also revealed positive correlations of *KPNA2* and *IMPAD1* with activated CD4 T cells, activated dendritic cells, and effector memory CD4 and CD8 T cells. In contrast, *DUSP9*, *CCNG1*, *CEP290*, and *GPR160* showed negative correlations with activated B cells, activated CD4 T cells, and effector memory CD8 T cells (Fig 4H).

### 3.5 Identifying key diagnostic genes using machine learning

**Fig 5:**
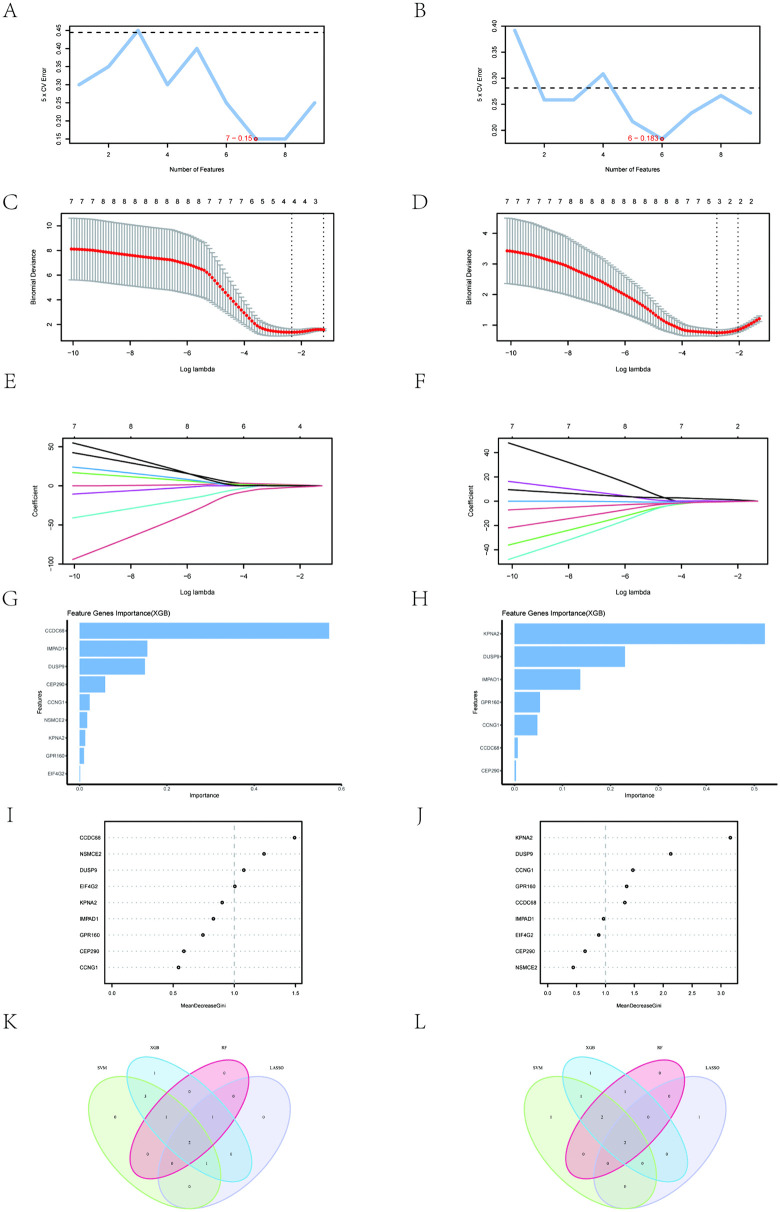
Screening key markers by machine learning. **(A)** Seven feature genes were screened by SVM-RFE algorithm for OSA. **(B)** Six feature genes were screened by SVM-RFE algorithm for IC/BPS. **(C,E)** Four feature genes were screened by LASSO algorithm for OSA. **(D,F)** Three feature genes were screened by LASSO algorithm for IC/BPS. **(G)** The top 10 genes in terms of importance as filtered by the XGBoost algorithm for OSA. **(H)** The top 10 genes in terms of importance as filtered by the XGBoost algorithm for IC/BPS. **(I)** Four feature genes were screened by RF algorithm. **(J)** Five feature genes were screened by RF algorithm. **(K)** Two key markers were screened by Venn diagram of four algorithms for OSA. **(L)** Two key markers were screened by Venn diagram of four algorithms for IC/BPS.

**Fig 6:**
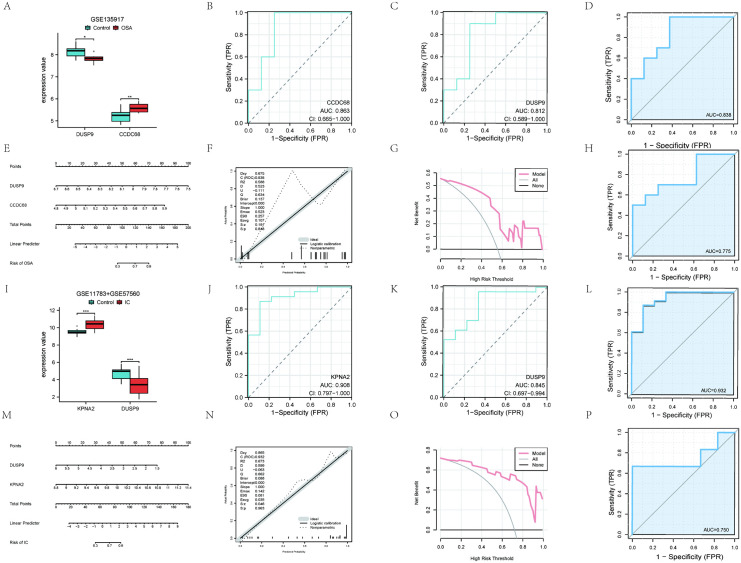
Candidate gene expression levels, diagnostic value and construction of nomograms. **(A)** Expression of DUSP9 and CCDC68 in GSE135917. **(B,C)** ROC curves of DUSP9 and CCDC68 genes in GSE135917. **(D)** Analysis of diagnostic models based on 2 genes in GSE135917. **(E)** Nomogram used to predict the risk of OSA. **(F)** Calibration curve of OSA. **(G)** DCA of OSA. **(H)** Validation of the OSA diagnostic model based on two genes in GSE38792. **(I)** Expression of DUSP9 and KPNA2 in GSE11783 and GSE57560. **(J,K)** ROC curves of DUSP9 and KPNA2 genes in GSE11783 and GSE57560. **(L)** Analysis of diagnostic models based on 2 genes in GSE11783 and GSE57560. **(M)** Nomogram used to predict the risk of IC/BPS. **(N)** Calibration curve of IC/BPS. **(O)** DCA of IC/BPS. **(P)** Validation of the IC/BPS diagnostic model based on two genes in GSE11783 and GSE57560.

Using the 9 key genes, we applied 4 machine learning algorithms to identify potential candidate genes related to OSA and IC/BPS (S10 and S11 Tables in S1 File). For OSA, we identified feature genes using four machine learning algorithms: 7 genes by SVM-RFE (Fig 5A), 4 by LASSO (Fig 5C and 5E), 9 by XGBoost based on importance (Fig 5G), and 4 by RF with relative importance exceeding 1.0 (Fig 5I). Venn diagram analysis shows 2 common genes across the four machine learning algorithms: *DUSP9* and *CCDC68* (Fig 5K). Similarly, for IC/BPS, we identified feature genes using four machine learning algorithms: 6 genes with the SVM-RFE algorithm (Fig 5B), 3 with LASSO (Fig 5D and 5F), 7 based on importance in XGBoost (Fig 5H), and 5 genes with relative importance exceeding 1.0 in the RF algorithm (Fig 5J). Subsequently, Venn diagram analysis revealed that there were 2 common genes among these algorithms: *DUSP9* and *KPNA2* (Fig 5L). In OSA patients, *DUSP9* was lower and *CCDC68* was higher than in the control group (Fig 6A); in IC patients, *DUSP9* was lower and *KPNA2* was higher than in the control group (Fig 6I). To further validate the robustness of these key biomarkers, we re-ran the analytical pipeline using a stricter threshold (|log2FC| > 0.3). Despite the reduction in input genes, *DUSP9* was consistently identified as a core shared gene across both OSA and IC/BPS through all analytical layers, confirming its reliability as a high-confidence biomarker (S12 Table, S3, S4 Figs in S1 File).

In addition, the ROC curve was calculated using five-fold cross-validation to evaluate the diagnostic performance of two core genes in the OSA test set. The results indicated that *DUSP9* and *CCDC68* could distinguish normal from OSA groups with AUCs of 0.812 and 0.863, respectively (Fig 6B and 6C). Using GSE13597 for training and GSE38792 for validation, the model achieved AUCs of 0.838 (Fig 6D) and 0.775 (Fig 6H). Similarly, in the IC/BPS test set, *DUSP9* and *KPNA2* distinguished normal from IC/BPS groups with AUCs of 0.845 and 0.908 (Fig 6J and 6K), respectively. GSE11783 and GSE57560 served as the training sets, and GSE11839 was used for external validation. The model achieved an AUC of 0.932 (Fig 6L) during training and 0.750 during validation (Fig 6P).

### 3.6 Construction of diagnostic models

To improve diagnostic and predictive accuracy, we constructed a nomogram using logistic regression analysis based on the central genes *DUSP9* and *CCDC68* (Fig 6E). The calibration plot shows that this nomogram model’s predictive ability closely matches the ideal model (Fig 6F). Furthermore, decision curve analysis indicates that decisions based on the nomogram model may assist in diagnosing OSA (Fig 6G). Similarly, another nomogram was constructed based on the central genes *DUSP9* and *KPNA2* (Fig 6M). For the OSA and IC/BPS model, the probability of disease was calculated using the logistic regression formula (S13 Table in S1 File). The calibration plot shows that this nomogram diagnostic model’s predictive ability is close to the ideal model (Fig 6N). Moreover, the decision quality assessment suggests that decisions based on the nomogram model may aid in diagnosing IC/BPS (Fig 6O).

### 3.7 Characteristics of core genes

**Fig 7:**
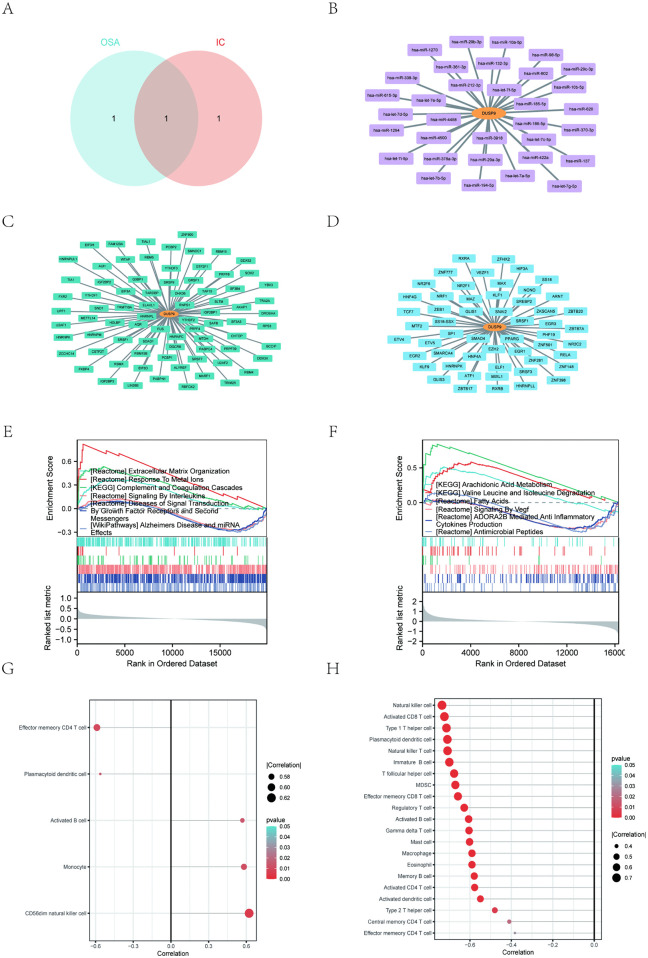
Characteristics of core genes and miRNA, RBP and TF interaction network. **(A)** One core gene were screened by Venn diagram of machine learning between OSA and IC/BPS. **(B)** Biomarker and miRNA interaction network. **(C)** Biomarker and RNA-binding proteins (RBPs) interaction network. **(D)** Biomarker-transcription factor (TF) interaction network. (E) GSEA pathway enrichment analysis associated with *DUSP9* for OSA. (F) GSEA pathway enrichment analysis associated with *DUSP9* for IC/BPS. (G) Lollipop chart of the correlation between *DUSP9* and immune cell types for OSA. (H) Lollipop chart of the correlation between *DUSP9* and immune cell types for IC/BPS.

Comparing the two candidate genes of OSA with those of IC/BPS, *DUSP9* is the only gene overlapping between the two subgroups (Fig 7A). To investigate *DUSP9*’s role in OSA and IC/BPS, we used GSEA. We found that certain pathways are crucial for OSA, including the complement and coagulation cascade, interleukin signaling, and growth factor receptor-mediated signaling (Fig 7E). In OSA patients, *DUSP9* correlated with immune cell infiltration, exhibiting the strongest positive link with CD56dim natural killer cells (R = 0.622) (Fig 7G). Regarding IC/BPS, GSEA highlighted pathways such as arachidonic acid metabolism, fatty acid metabolism, and VEGF signaling as key contributors (Fig 7F). *DUSP9* correlated with immune cell infiltration, showing the strongest negative correlation with natural killer cells (R = −0.735) (Fig 7H).

### 3.8 Biomarkers and miRNA, RBP and TF action networks

We used the Starbase database to construct a *DUSP9*-related gene network. This network includes mRNA and miRNA, as well as *DUSP9* mRNA and RNA-binding proteins (RBPs). Specifically, the network includes one *DUSP9* mRNA interacting with 33 miRNAs (Fig 7B) and 79 RBPs (Fig 7C). Additionally, using the ChIPBase v3.0 database, we constructed a *DUSP9* mRNA-transcription factor network consisting of *DUSP9* mRNA and 54 transcription factors (Fig 7D).

## 4 Discussion

OSA and IC/BPS are chronic diseases that severely affect patients’ quality of life. Extensive literature suggests they may share common pathophysiological mechanisms, including inflammatory response, oxidative stress, and neuroendocrine regulation. This study aims to investigate the potential associations and molecular mechanisms between these two diseases through bioinformatics analysis and machine learning methods, providing a theoretical basis for developing new diagnostic tools and treatment strategies.

This study uses bioinformatics analysis combined with machine learning methods to identify potential biomarkers associated with OSA and IC/BPS. Through differential expression analysis of gene expression data for OSA and IC/BPS, 93 common differentially expressed genes were identified. While functional enrichment analysis showed enrichment in various processes, our core finding is the identification of key genes such as *DUSP9*, *CCDC68*, and *KPNA2* through machine learning algorithms. Based on these genes, a diagnostic model was constructed, which demonstrated strong predictive accuracy in distinguishing OSA or IC/BPS patients from healthy controls.

The exploration of potential shared mechanisms is supported by extensive existing literature, which provides a framework for interpreting our genomic findings. Inflammation is a common pathological feature of both OSA and IC/BPS [8,9]. Pro-inflammatory factors such as TNF-α and IL-6 are significantly elevated in OSA patients [10]. These factors not only contribute to the occurrence and development of cardiovascular diseases but may also affect the urinary system through the bloodstream [11]. This process can promote bladder inflammation and exacerbate the symptoms of IC/BPS [7]. Functional enrichment analysis from our data indicated that inflammation-related signaling pathways are significantly enriched, including the RIG-I-like receptor signaling pathway, NOD-like receptor signaling pathway, cytokine-cytokine receptor interaction, and JAK-STAT signaling pathway. Activation of these pathways may cause excessive inflammatory factor production, leading to both local and systemic inflammation [12,13]. Furthermore, immune cell infiltration analysis reveals significant differences in the immune cell composition between OSA and IC/BPS patients. Notably, NK cells, effector memory CD4 + T cells, and effector memory CD8 + T cells show distinct variations. The excessive infiltration of these immune cells may worsen inflammation, creating a self-perpetuating cycle [14,15]. The key gene *DUSP9*, identified through our machine learning approach, plays a key role in regulating the inflammatory response, and its downregulation may abnormally activate the MAPK signaling pathway, thereby worsening the inflammatory response [16]. Our immune infiltration analysis indicates that *DUSP9* expression correlates with NK cell infiltration, suggesting that *DUSP9* may modulate the inflammatory process by regulating immune cell activity.

Similarly, the role of oxidative stress is well-documented in both disorders [17,18]. Patients with OSA experience repeated hypoxic and awakening events. These events significantly increase oxidative stress levels, elevating intracellular reactive oxygen species (ROS) and reactive nitrogen species (RNS) [19]. These reactive molecules further harm cell membranes, proteins, and DNA, triggering inflammatory responses and subsequent tissue injury [20]. In IC/BPS, oxidative stress may promote the release of inflammatory factors by activating inflammatory signaling pathways (such as NF-κB and MAPK pathways), damaging bladder epithelial cells, causing dysfunction of the bladder epithelial barrier, and increasing the bladder’s sensitivity to irritants, thereby triggering symptoms such as pain and urinary frequency [21]. Additionally, oxidative stress may increase sensory nerve fiber sensitivity by directly activating these fibers, which leads to excessive transmission of pain signals [22]. Previous studies have shown that *DUSP9* plays an important role in regulating the oxidative stress response. Its downregulation may lead to abnormal activation of the MAPK signaling pathway, which exacerbates oxidative damage to cells [23,24]. Additionally, it intensifies inflammatory responses and oxidative stress by affecting immune cell function [23]. Our findings, which highlight *DUSP9*’s centrality through machine identification, further suggest that its downregulation may exacerbate oxidative stress, affect immune cell function, and sensitize nerve fibers, thereby further aggravating the symptoms of these diseases. This discovery not only provides a new perspective for understanding the pathophysiological connection between OSA and IC/BPS, but it also offers potential targets for developing therapeutic strategies against oxidative stress.

Finally, substantial evidence implicates neuroendocrine dysregulation as a key connecting pathway. Patients with OSA often show excessive activation of the sympathetic nervous system [25]. They also frequently experience dysfunction of the HPA axis [26]. These neuroendocrine changes affect inflammatory responses and immune cell function, and they also lead to psychological changes such as anxiety, depression, and stress [27]. Psychological stress activates the HPA axis and sympathetic nervous system, triggering the release of corticotropin-releasing hormone (CRH) and cortisol, which regulate inflammatory responses and immune cell distribution [28]. For example, CRH can activate mast cells to release inflammatory mediators like histamine, leukotrienes, and serotonin, which contribute to neurogenic inflammation [29]. Additionally, psychological stress may also lead to an increase in nerve fiber density, further exacerbating bladder dysfunction [30]. Therefore, neuroendocrine regulation may play a crucial role in inflammatory responses and immune cell infiltration in OSA and IC/BPS. It may also worsen disease symptoms by affecting psychological states [31,32].

This study used bioinformatics analysis and machine learning methods to identify potential biomarkers linked to OSA and IC/BPS. Our primary contribution is the identification of *DUSP9* as a key connecting gene, which may help explain potential shared pathological mechanisms between these two diseases. These findings not only provide important molecular evidence for understanding the relationship between OSA and IC/BPS but also offer new targets and strategies for future diagnosis and therapy. Notably, the identification of *DUSP9* presents possibilities for developing new diagnostic tools and treatment plans. The expression level of *DUSP9* can be used for the early diagnosis of OSA and IC/BPS and can help assess treatment efficacy. Furthermore, *DUSP9* and its regulated MAPK signaling pathway can serve as targets for developing new therapeutics to improve patient prognosis and quality of life.

This study successfully screened potential biomarkers related to OSA and IC/BPS using bioinformatics and machine learning. However, some limitations remain. First, the relatively small sample size may reduce the statistical power and limit the clinical reliability of the results. Moreover, the lack of wet lab validation limits in-depth functional studies of key genes and pathways. Furthermore, the incomplete availability of detailed clinical metadata, including individual-level OSA severity metrics (e.g., AHI) and standardized IC/BPS diagnostic criteria or symptom scores, may constrain the precision of our phenotypic correlations and disease stratification. Additionally, we acknowledge that the use of transcriptomic data derived from different tissue sources (adipose tissue for OSA and bladder tissue for IC/BPS) may introduce bias due to tissue-specific gene expression patterns. However, our approach is justified by the systemic nature of both diseases, where potential shared pathological mechanisms can manifest across multiple tissues. The identification of *DUSP9* as a key gene in both contexts supports its potential role in systemic responses, and our findings are framed as an exploration of systemic-local links. Our study is limited by the inherent constraints of the source genomic datasets, which lack detailed clinical annotations such as apnea-hypopnea index (AHI) for OSA severity and standardized diagnostic criteria or symptom scores for IC/BPS. This precluded stratification by disease severity or adjustment for key clinical confounders. To ensure transparency, we have compiled all accessible individual-level clinical data in S1 Table in S1 File. Future research should validate these biomarkers in prospectively recruited, deeply phenotyped cohorts that include standardized severity metrics and comprehensive clinical histories. In addition, combining in vitro experiments and animal models to explore *DUSP9* mechanisms in OSA and IC/BPS will aid in developing targeted therapies and offer deeper insights into its role.

## 5 Conclusion

This study integrates bioinformatics and machine learning to identify *DUSP9* as a key gene connecting OSA and IC/BPS, with functional analyses further suggesting involvement of shared pathological processes in inflammation, oxidative stress, and neuroendocrine regulation. These results provide important molecular insights into the link between these two disorders and highlight *DUSP9* as a promising candidate for developing diagnostic biomarkers and targeted therapies. Future studies should prioritize experimental validation of *DUSP9*’s functional role and its translation into clinically applicable tools.

## Supporting information


S1 File.
**S1 Table.** The expression matrix and grouping information of GSE135917 (OSA). **S2 Table.** The expression matrix and grouping information of GSE11783 and GSE57560 (IC/BPS). **S3 Table.** The differential expression genes of OSA and IC/BPS. **S4 Table.** Differential expression genes using stricter threshold (|log2FC| > 0.3). **S5 Table.** GO/KEGG enrichment analysis of DEGs. **S6 Table.** GSEA analysis on OSA and IC/BPS samples. **S7 Table.** The overlapping genes between the strongest positive modules of OSA and IC/BPS. **S8 Table.** The overlapping genes between WGCNA module genes and DEGs. **S9 Table.** GO enrichment analysis of the nine overlapping genes between WGCNA module genes and DEGs. **S10 Table.** Key markers by machine learning of OSA. **S11 Table.** Key markers by machine learning of IC/BPS. **S12 Table.** Integration of WGCNA and machine learning results using stricter threshold (|log₂FC| > 0.3). **S13 Table.** The risk calculation formula for the OSA and IC/BPS diagnostic model. **S1 Fig.** Identification of soft-threshold power based on GSE135917 (OSA). **S2 Fig.** Identification of soft-threshold power based on GSE11783 and GSE57560 (IC/BPS). **S3 Fig.** Venn diagram of machine learning key gene overlap for IC/BPS under the stricter threshold (|log2 FC| > 0.3). **S4 Fig.** Venn diagram of machine learning key gene overlap for OSA under the stricter threshold (|log2 FC| > 0.3).
(ZIP)
